# Monitoring of Pregnant Women Using the “Risk Identification, Evaluation Counseling, Systematic Monitoring, Troubleshooting” (REST) Mobile App: Protocol for a Cluster Randomized Controlled Trial

**DOI:** 10.2196/66774

**Published:** 2025-08-06

**Authors:** Restu Pangestuti, Prima Dhewi Ratrikaningtyas, Adi Heru Sutomo

**Affiliations:** 1 Doctorate Program of Medical and Health Science Faculty of Medicine, Public Health, and Nursing Universitas Gadjah Mada Yogyakarta Indonesia; 2 Department of Midwifery Faculty of Health Sciences Alma Ata University Yogyakarta Indonesia; 3 Department of Biostatistics, Epidemiology and Population Health Faculty of Medicine, Public Health, and Nursing Universitas Gadjah Mada Yogyakarta Indonesia; 4 Faculty of Medicine, Public Health, and Nursing Universitas Gadjah Mada Yogyakarta Indonesia

**Keywords:** pregnant woman, midwife, antenatal screening, mobile health, mHealth, application, app, randomized controlled trial, RCT, antenatal class, risk identification, evaluation counseling, systematic monitoring

## Abstract

**Background:**

The maternal mortality rate (MMR) in Indonesia is still quite high and has not reached the national target. The success of maternal health programs can be assessed through the main indicator of the MMR. Pregnancy monitoring is one of the efforts to reduce the increase in the MMR.

**Objective:**

This study aims to improve maternal and child safety and health during pregnancy and childbirth through pregnancy monitoring using a mobile app called REST (Risk Identification, Evaluation Counseling, Systematic Monitoring, Troubleshooting).

**Methods:**

The research used the cluster randomized controlled trial (RCT) design, involving pregnant women from 11 subdistricts in Purworejo Regency, who were randomized to 11 clusters in the intervention group and 11 clusters in the control group. The 22 ANC class clusters comprised 22 midwives and 220 pregnant women. The intervention group received monitoring using the REST mobile app, while the control group received standard pregnancy monitoring in the antenatal care (ANC) class. The mentoring program included the use of the REST mobile app, which consists of pregnancy checks according to the 10T pregnancy examination standard. The app was used by midwives and pregnant women, starting from the second trimester of pregnancy to childbirth.

**Results:**

In July 2023, the REST mobile app was prepared and tested in small community groups, including midwives and pregnant women, through simulation, and in September 2023, the app was further refined based on feedback from these groups and introduced to study participants. The majority of participants were aged 20-35 years (n=168, 76.4%), consistent with national demographic trends. Approximately 185 (84.1%) had secondary education (junior and senior high school), and 57 (25.9%) had tertiary education (college graduates). The participants were evenly distributed across economic quintiles, reflecting diverse socioeconomic backgrounds, and most lived approximately 1 km from a health facility. Ethical approval was obtained in April 2024. Staff training was conducted from July to October 2023. Participants were recruited from November 2023 to January 2024, the intervention was implemented from February to July 2024, and data were collected from August 2024 to February 2025. Data were analyzed in April 2025, and dissemination of results is expected by the end of 2025.

**Conclusions:**

Pregnancy monitoring using the REST mobile app is expected to have a significant influence on the number of ANC visits, reduce pregnancy complications, improve normal delivery methods, and ensure the birth weight of the baby stays within normal limits (≥2500 g).

**Trial Registration:**

ClinicalTrials.gov NCT05741931; https://clinicaltrials.gov/study/NCT05741931

**International Registered Report Identifier (IRRID):**

DERR1-10.2196/66774

## Introduction

The success of maternal health programs can be assessed through the main indicator of the maternal mortality rate (MMR). The gestation period is the focus of the main problem in preventing high MMRs, as it is during this time that the majority of risk factors for adverse maternal and fetal outcomes emerge. In particular, nutritional and metabolic issues, such as hypocalcemia, have been associated with complications in pregnancy, especially in resource-limited settings [1-3]. The gestation period is the focus of the main problem in preventing high MMRs. The World Health Organization (WHO) recommends that health workers improve consultation and care for pregnant women [4].

Antenatal care (ANC) services in normal pregnancy are provided at least 6 times: 2 times in the first trimester, 1 time in the second trimester, and 3 times in the third trimester. ANC examinations in normal pregnant women are carried out by a doctor at least 2 times in the first and third trimesters [5]. Failure in pregnancy care or ANC usually occurs due to social and economic factors, such as low education and family income, being too young or too old, living far from health facilities, unwanted pregnancy, and the consumption of illegal drugs [4].

The MMR in Central Java Province in 2021 was 199 per 100,000 live births (1011 cases), an increase compared to the MMR in 2020 of 98.6 per 100,000 live births (530 cases) and in 2019 of 76.93 per 100,000 live births (416 cases). Cases of maternal mortality increased from 2019 onward. The cause of disruption in maternal and child health services during the pandemic was due to major changes in health facility services and public health [6]. The still high mortality rate shows that the quality of maternal and neonatal services is not optimal. The issue of “3 late” is still the main problem in maternal health services, namely late decision-making, late arrival at the referral point, and late getting the services needed at health facilities. The low quality of pregnancy examination services is also reflected in the low compliance with ANC standards, which include 10 pregnancy examinations [7].

There are several studies on pregnancy using mobile apps, such as the m4Change app [8], which contains health promotion around maternal and child health, with the results of improving the quality of ANC. The PANDA (Pregnancy and Newborn Diagnosis Assessment) app is used to monitor during ANC, with the results of knowing the cause of pregnancy complications [9]. The Innovative Mobile Phone Technology for Community Health Operations (ImTeCHO) app for scheduling home visits, screening for complications, and counseling has effective results in areas that are difficult for health facilities to reach [10]. This mobile app can be used as a medium by pregnant women to monitor their pregnancy and by midwives who conduct pregnancy checks. Mobile apps should be used by considering their effectiveness and quality of use.

Pregnancy monitoring is a decision-making action for pregnant women and their fetuses in order to overcome previous adverse events in fetal development. Pregnancy monitoring facilitates early discovery of abnormal developments and complications and supports timely intervention so that early treatment can be administered and disorders and risks for pregnant women reduced. If pregnancy disorders are found in the mother and fetus, efforts are made to reduce the high risk in pregnancy [11].

Pregnancy monitoring is one of the efforts made by both health workers and health cadres in monitoring the condition of both mother and fetus during pregnancy, as it plays a critical role in early detection of complications and improving maternal outcomes. Monitoring by health care providers during the gestation period, such as to provide accurate real-time data on how many pregnant women are enrolled in ANC and what the ANC class characteristics, interventions, and outcome approaches are, will improve ANC information systems that can reduce maternal mortality cases [12].

An assessment of the implementation of health services for pregnant women can be carried out by looking at the K1, K4, and K6 coverage. K1 coverage is the number of pregnant women who have received ANC services for the first time by health workers compared to the target number of pregnant women in 1 work area within a period of 1 year. K4 coverage is the number of pregnant women who have received ANC services in accordance with the standard at least 4 examinations according to the recommended schedule in each trimester compared to the target number of pregnant women in 1 work area within 1 year. K6 coverage is the number of pregnant women who have received ANC services in accordance with the standard of at least 6 examinations and at least 2 doctor’s examinations according to the recommended schedule in each semester compared to the target number of pregnant women in 1 work area within a period of 1 year. This indicator shows access to health services for pregnant women and the level of compliance of pregnant women in checking their pregnancy with health workers [5]

The ANC class is an effort by the Government of Indonesia to promote health and prevent maternal and infant mortality. The Government of Indonesia began implementing the ANC class program in 2009. The program aims to increase the understanding of pregnancy, the use of ANC and postpartum family planning services, and awareness of infectious diseases. The target population of the ANC class is mothers with a gestational age of 22-36 weeks. Each class consists of a maximum of 10 pregnant women and is facilitated by midwives or health workers who have received training in ANC [13]. Mobile apps have been widely used by various groups, such as health cadres, health service users, and health service providers, to facilitate activities in the health sector [14].

Mobile apps for health are often used to improve the quality of health services. Mobile apps can continue to evolve through the correction of errors adjusted to needs [15]. There are many variations of mobile apps used for health purposes. They can contain patient medical records, health consultations, and health training, as well as health promotion [16,17]. Pregnant women make up a significant proportion of the world’s population. With an average age of 30 years, pregnant women are a generation that wants to get to know new technologies and expand medical care in the digital sector. There are many types of mobile apps used in monitoring the health of pregnant women. These apps are often used in the field of midwifery because they are considered appropriate and easy to implement. They are used not only for pregnant women but also for several groups involved, including couples and families. Pregnancy-related mobile apps for postnatal care and parenting have also been developed. The success in modifying a pregnancy-related app and complying with its use make it a new lifestyle intervention for users [18].

Based on the aforementioned findings, it is important to conduct research on the use of mobile apps for pregnancy monitoring. The objectives of the study are to check for the presence of pregnancy complications, compare the intervention and control groups in the context of the number of pregnant women who give birth normally, ensure that the birth weight of babies reaches ≥2500 g, and improve the number of ANC visits to ≥6.

The benefits achieved from the results of the study are making it easier (1) for pregnant women monitor their pregnancy conditions and (2) for village midwives to be able to assist in monitoring maternal health during pregnancy, recognizing maternal problems, and helping make timely referrals. The benefits for related agencies are input for public health centers, the independent practice of midwives and doctors, the Health Office, and the regional general hospital in monitoring pregnant women to prevent maternal and infant mortality. The benefits for researchers include motivating and facilitating community leaders, cadres, and communities, as well as increasing cross-sector cooperation in monitoring the health of pregnant women. For science, conducting pregnancy monitoring can be a novelty.

## Methods

### Study Design

This research is an experimental study with a cluster randomized controlled trial (RCT) design. Cluster RCT is a research design that has multilevel characteristics. Generally, cluster RCT research uses 2 levels, the cluster or group level and the individual level. In this study, the class cluster will include pregnant women and participants of the ANC class. To minimize the risk of selection bias, we implemented the following strategies: (1) randomization at the cluster level was performed after cluster enrollment, and baseline characteristics of participants across the intervention and control groups will be carefully assessed and reported. The research design is described in Table 1.

**Table 1:** Research design.

Group	Activity sequence 1			Activity sequence 2			Activity sequence 3			Activity sequence 4
	Observation	Intervention	Observation		Intervention	Observation		Intervention	Observation	
Control	O_0_^a^	X_0_^b^	O_1_^c^		X_0_	O_2_^d^		X_0_	O_3_^e^	
Intervention	O_0_	X_1_^f^	O_1_		X_2_^g^	O_2_		X_3_^h^	O_3_	

^a^O_0_: early identification in pregnant women in the first class of pregnant women.

^b^X_0_: standard intervention for routine antenatal classes without an app.

^c^O_1_: observations in the second class of pregnant women (trimester 2).

^d^O_2_: observations in the third class of pregnant women (trimester 3).

^e^O_3_: observation of childbirth and the newborn.

^f^X_1_: introduction of the REST (Risk Identification, Evaluation Counseling, Systematic Monitoring, Troubleshooting) mobile app for pregnancy monitoring for mothers and midwives using the 10Tpregnancy examination standard.

^g^X_2_: monitoring pregnant women after using the mobile app.

^h^X_3_: monitoring pregnant women using the mobile app and preparing for childbirth during the third trimester.

Assessment of the results of the research will begin from the implementation of the first class of pregnant women. The total number of meetings was 3. The final result (a comparison of the expected outcome) was at the time of delivery. The research was carried out after ethical approval was obtained. The research locations are in 11 subdistricts in Purworejo Regency (Central Java, Indonesia): Loano, Purwodadi, Bruno, Butuh, Purworejo, Bagelen, Kaligesing, Kemiri, Kutoarjo, Ngombol, and Pituruh. These 11 subdistricts were chosen because they have health centers with inpatient services that make it easier for pregnant women to give birth in their areas.

Each ANC class group consisted of 10 pregnant women and 1 midwife. In each of the 3 meetings, an examination based on the 10T pregnancy examination standard was conducted, which included measuring the weight and height (T1), blood pressure (T2), mid-upper arm circumference (T3), uterine fundal height (T4), tetanus immunization (T5), iron supplement tablets (T6), fetal presentation and fetal heart rate (T7), counseling (including childbirth and postpartum preparation; T8), hemoglobin levels (T9), and care as needed (T10), as per the policy of the Indonesian Ministry of Health [5]. The results of the examination at each meeting were entered into the REST (Risk Identification, Evaluation Counseling, Systematic Monitoring, Troubleshooting) mobile app, and the pregnant women could see these results. If they performed a self-examination outside the ANC class schedule, they could fill in the results of the examination according to what they knew. Midwives and pregnant women were provided with training and a guidebook module for using the app. The control group carried out the standard ANC class without the use of the app, and they were exempt from recording examination results.

### Population

The target population in this study was all pregnant women in Purworejo Regency. The affordable population was all pregnant women in 11 subdistricts. In total, 14 inpatient health centers were included, which included 22 clusters of ANC classes. Each cluster or class group comprised 10 pregnant women who met the inclusion criteria.

The inclusion criteria were as follows: pregnant women with a gestational age of 20-22 weeks (trimester 2), pregnant women who had a pregnancy examination at a health facility, mothers who planned to stay in the research area for at least the next 2 years, and mothers who were willing to participate in the research by signing an informed consent form.

The exclusion criteria were as follows: chronic diseases that require special pregnancy care, inability to give birth normally, and inability to operate an Android cellphone. The applicant was dropped if she moved outside the research area, could not be contacted after agreeing to provide informed consent, or decided to stop participating in the research.

### Sample Size

The sample size for this study was determined based on the following formula of Lemeshow [19]:

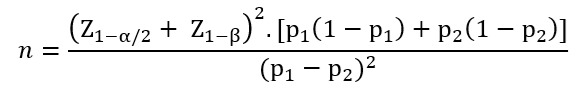



where n is the number of samples per group, Z_1–α/2_=1.96 (at the level of 95% trust), Z_1–β_=0.842 (at 80% power), p_1_ is the proportion of outcomes in the intervention group, p_2_ is the proportion of outcomes in the control group, and p_1_ – p_2_ is the difference between the 2 proportions to be compared.

Based on the calculation of the sample size from previous studies [4,20-22], p_1_=0.92 and p_1_=0.72, so the minimum sample size for the intervention and control groups was 54 respondents each. In cluster RCT research, it is necessary to consider the *design effect* (DE) in determining the sample size. The formula used is DE = 1 + (m – 1), where m=10 (the average number of samples per cluster) and is the intraclass correlation coefficient, assumed to be 0.05.

Based on the calculation results, a DE of 1.45 was obtained. Furthermore, the sample size that was obtained was multiplied by the DE, resulting in a sample size of 78.3, which was rounded to 79 respondents per group. Therefore, the total number of participants needed was 158, although considering a loss to follow-up of ~35% (based on a preliminary study of the attendance of ANC classes in 2019 in Purworejo Regency)*.* Therefore, it was necessary to take a total of 214 (107 participants in the intervention and the control group each) at the beginning of the study through cluster sampling. The number of clusters to be used was 22 groups of pregnant women, so each group needed 9.72 samples, rounded 10 pregnant women in each group.

### Randomization, Allocation, and Blinding

Sample determination was carried out using a cluster sampling approach. The target population was all inpatient health centers spread across 11 subdistricts in Purworejo Regency. Based on data from the Health Office, 14 inpatient health centers met the inclusion criteria. Of these, 11 (78.6%) were randomly selected, each representing 1 subdistrict. These 11 subdistricts consisting of 22 pregnant class clusters (C1-C22) were randomized into an intervention group (n=11, 50%) and a control group (n=11, 50%) using the compute-generated random allocation method. A list of cluster codes was entered, and a random sequence was used to divide the groups in order. The results of randomization determined that the first 11 clusters in the list were allocated to the intervention group (C1-C11), while the remaining 11 clusters were allocated to the control group (C12-C22). Randomization was carried out by independent researchers to ensure unbiased random allocation.

The group allocation was kept secret until the intervention group received treatment. Blinding was not possible both for the treatment provider (researcher) and for the sample because the monitoring intervention allowed for active interaction between treatment provider and sample. To ensure the accuracy and completeness of the report, this study adheres to the standards set out in CONSORT (Consolidated Standards of Reporting Trials) EHEALTH V1.6 checklist (Multimedia Appendix 1), which includes a detailed description of the digital intervention and research methodology [23]. The timeline of participant participation was developed in accordance with the SPIRIT (Standard Protocol Items: Recommendations for Interventional Trials) statement (Table 2 and Multimedia Appendix 2) [24].

**Table 2:** SPIRIT^a^ checklist–recommended schedule for study participation.

Study period and activities		Enrollment time point	Allocation time point	Postallocation time points				Close-out time point
		Week 1	Week 0	Month 1	Month 2	Month 3	After month 3	
**Enrollment**								
	Informed consent	X^b^	—^c^	—	—	—	—	
	Eligibility screening	X	—	—	—	—	—	
	Random allocation (intervention vs control)	—	X	—	—	—	—	
**Interventions**								
	REST^d^ app installation and training^e^	X	—	—	—	—	—	
	REST mobile app usage by pregnant women and midwives^e^	—	—	X	X	X	X	
	Routine ANC^f^ class^g^	—	—	X	X	X	—	
**Assessments**								
	Baseline clinical measurements	—	—	X	—	—	—	
	ANC visit	—	—	X	X	X	—	
	Pregnancy complications	—	—	X	X	X	—	
	Mode of delivery	—	—	—	—	—	X	
	Birth weight	—	—	—	—	—	X	

^a^SPIRIT: Standard Protocol Items: Recommendations for Interventional Trials.

^b^Applicable.

^c^Not applicable.

^d^REST: Risk Identification, Evaluation Counseling, Systematic Monitoring, Troubleshooting.

^e^Intervention group.

^f^ANC: antenatal care.

^g^Intervention and control groups.

### Intervention

The REST mobile app provides pregnant women with real-time access to test results during pregnancy, personalized health reminders, and interactive communication with midwives, empowering them to make informed health decisions immediately. To minimize delays in reaching care, the app includes a birthing site–planning feature, helps midwives direct when urgent referrals are needed, and facilitates mobilization in the case of an emergency. To address delays in receiving adequate care in health care facilities, REST supports midwives by enabling structured antenatal monitoring and facilitating prereferral communication with referral centers, ensuring that health care facilities are better prepared to receive and manage incoming patients. The REST mobile app, designed for pregnancy monitoring from early pregnancy to delivery, has been officially copyrighted by the Ministry of Law and Human Rights of the Republic of Indonesia (ID: 000655305).

Before starting the intervention, the midwives were first given face-to-face training on the use of apps and procedures for implementing the ANC class. This joint intervention was deemed necessary to ensure consistent use of the platform during the trial. Next, training was continued for the participants of the ANC class regarding how to use and fill in the app. A handbook for using the app was also provided to all participants in the intervention group. Participants in the control group received standard ANC class services, as outlined by Indonesia’s national maternal health program. These services include providing health education, nutrition counseling, and pregnancy gymnastics activities in accordance with meeting materials in ANC classes conducted by midwives. No digital tools or mobile apps were introduced to this group. Educational materials were delivered in print format as per standard practices, and no structured training or technological support was provided. This approach ensured that any differences in outcomes observed between the control and intervention groups could be attributed to the use of the REST mobile app.

The intervention that was implemented was in the form of a Template for Intervention Description and Replication (TIDieR), as shown in Table 3 [25].

**Table 3:** The TIDieR^a^ format.

Item number	Item name	Description
1	Intervention	Use of the REST^b^ mobile app
2	Reasons for the intervention	Lack of discipline for pregnant women to participate in government program activities that support pregnancy health, such as ANCc classes. The ANC class is a monitoring effort to improve the welfare of mothers and babies. Monitoring using the REST mobile app is expected to make it easier for pregnant women and midwives to monitor the health condition of mothers.
3	Action	Providing assistance to mothers during pregnancy using mobile apps. Assistance is provided to monitor the health condition of both mother and fetus. The mother takes part in a class for pregnant women and is examined (10 pregnancy examinations: height and weight, blood pressure, mid-upper arm circumference, uterine fundal height, tetanus immunization, iron supplement tablets, fetal presentation and fetal heart rate, counseling, laboratory tests, and case management).
4	Procedure	Conducting initial identification of mothers at the first meeting of the ANC class. Mothers are examined by midwives at every class meeting of pregnant women, and the results of the pregnancy examination are entered into the REST mobile app. Each mother gets a different evaluation from the midwife according to the results of the mother’s pregnancy examination. The ANC class is held 3 times, with a duration of approximately 90 minutes per meeting. In each implementation of the class for pregnant woman, the mother is given pretest and posttest questions.
5	Organizers	Principal investigator: ensures the readiness of activities and the completeness of the equipment needed during the ANC class. Midwife facilitator (trained in the use of mobile apps): carries out examinations of pregnant women, provides counseling, and documents examination results into mobile apps. Cadres (trained in the measurement of height, weight, and blood pressure): assist midwives during the implementation of classes for pregnant women. Enumerator (trained in the use of mobile apps): assists the principal investigator in monitoring the progress of ANC class activities.
6	Intervention delivery model	The provision of interventions was carried out face to face in each group of pregnant women. Pregnant women were required to attend classes 3 times and were also advised to fill in the results of examinations outside of the class activities so that the midwives could still monitor.
7	Venue and facilities	Pregnancy monitoring was carried out in the Purworejo Regency area. The infrastructure used was stationery, mobile phones (smartphones), weight scales and height-measuring instruments, tools to check hemoglobin levels, dopplers to check the fetal heart rate, mid-upper arm circumference tape, metline to measure the uterine fundal height, digital sphygmomanometers, and research questionnaires.
8	Changes or modifications during the process	The time of the class for pregnant woman was adjusted for the mothers and midwives according to an agreement. Pregnancy monitoring using the REST mobile app was adjusted to local conditions and the ownership of mobile phones by pregnant women. Furthermore, pregnancy monitoring using an app in the ANC class was carried out by midwives at the workplace or their respective practices.
9	Planning	On the REST mobile app, a reminder was created to remind midwives and mothers of the date of the ANC class. This reminder appeared in the form of a notification the day before the class for pregnant women was held.

^a^TIDieR: Template for Intervention Description and Replication.

^b^REST: Risk Identification, Evaluation Counseling, Systematic Monitoring, Troubleshooting.

^c^ANC: antenatal care.

The intervention given to the treatment group used the REST mobile app. The app was validated by 3 experts: an obstetrician and gynecologist, a health application expert, and a community midwifery expert. The app was piloted in a small group of pregnant woman communities who were not involved in the intervention study. Evaluation included the format, substance, presentation, and language. The results of the trial were measured by the distribution of simple questionnaires, interviews, and observations. After revision for refinement, the app was ready to use in research.

In a preimplementation survey involving 25 pregnant women and 10 midwives in Purworejo Regency, the REST app scored an average of 79.3 on the System Usability Scale (SUS), indicating above-average usability. Participants found the navigation intuitive, the language clear, and the visual design attractive. Of the 25 pregnant women, 23 (92%) successfully downloaded and used the REST mobile app independently within 3 days of onboarding. Midwives reported that training sessions only took an average of 60 minutes, and no major technical hurdles were encountered. In total, 30 (85.7%) pregnant women and midwives reported being very satisfied with the app and recommended it for use in pregnancy monitoring. To ensure cultural sensitivity and responsiveness to the needs of the target population, the study was developed in close collaboration with local stakeholders, including community health workers, midwives, and representatives of pregnant women in the study area. The content and language used in the REST mobile app were adapted to reflect local cultural norms, health beliefs, and communication styles, using Bahasa Indonesia and regionally appropriate terminology. Prior to implementation, the app and data collection instruments were pilot-tested with a sample of the target population to assess clarity, relevance, and acceptability.

**Figure 1:**
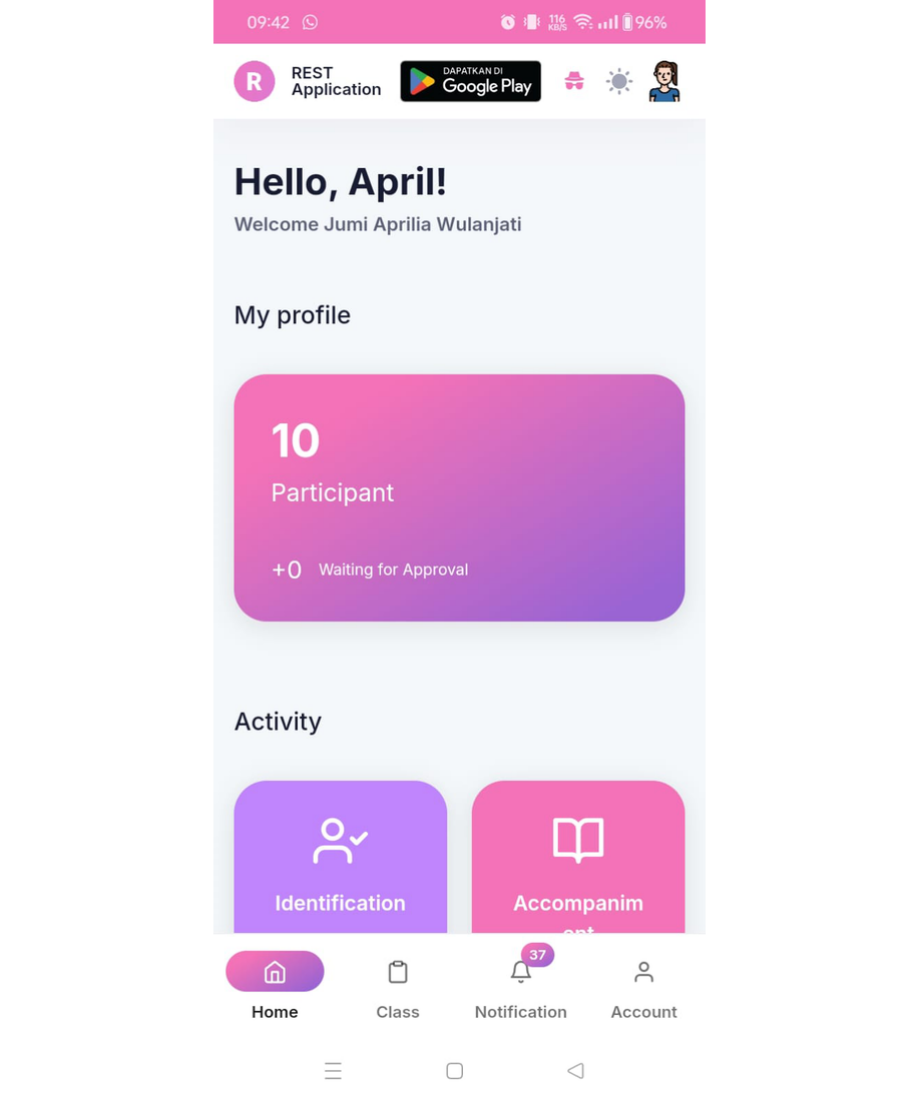
Image of the REST mobile app's welcome screen. REST: Risk Identification, Evaluation Counseling, Systematic Monitoring, Troubleshooting.

**Figure 2:**
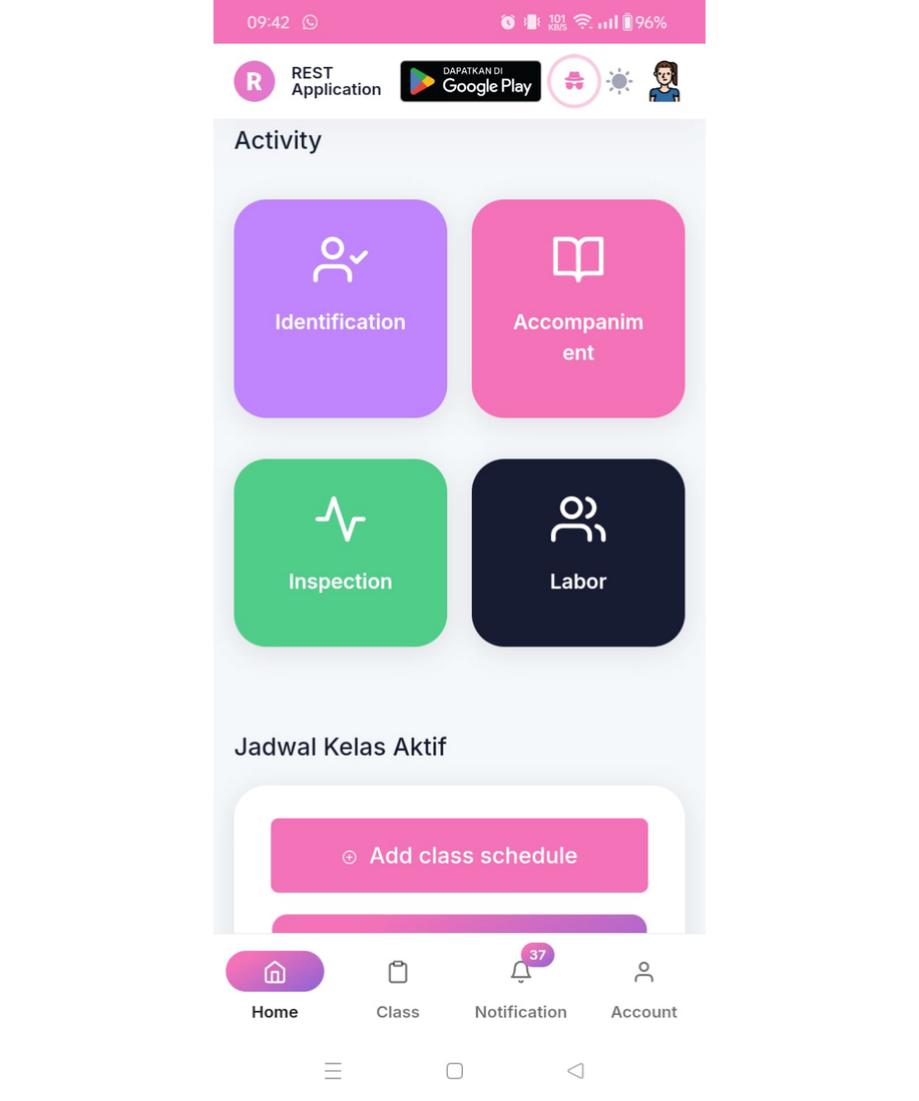
Image of the REST mobile app's activity screen. The midwife can fill in the data of the results of the mother's examination during the implementation of the ANC class, and the mother can see it in pregnant women's app version. ANC: antenatal care; REST: Risk Identification, Evaluation Counseling, Systematic Monitoring, Troubleshooting.

**Figure 3:**
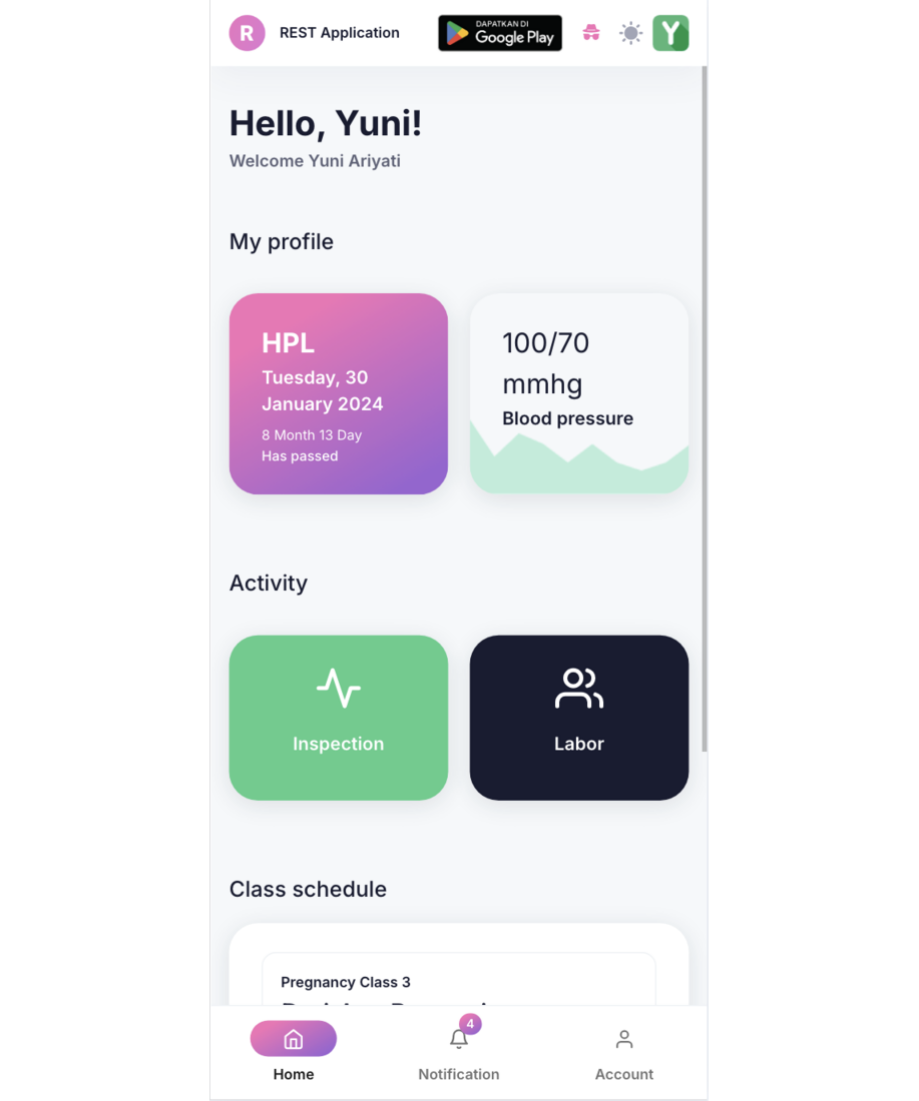
Image of the REST mobile app's profile screen. Pregnant women (participants) can monitor their pregnancy based on the results of the examination that have been input by the facilitator midwife and can also fill in the results of the examination carried out outside the implementation of the ANC class. ANC: antenatal care; REST: Risk Identification, Evaluation Counseling, Systematic Monitoring, Troubleshooting.

Figures 1-3 show images of the validated and tested app [26]. The app display for midwives is different from that for participants; in the version for midwives, detailed data of pregnant women who are members of the ANC class group appear, monitored by each midwife.

The fidelity of the intervention was assessed through a variety of strategies. First, the REST mobile app included an internal analytics system that automatically recorded user activities, such as examination results filled in by midwives and the completion of scheduled tasks by pregnant women (recording data on ANC visits). Second, the research assistant conducted periodic monitoring during ANC classes using a standard checklist to observe whether midwives and pregnant women were using the app according to protocol. Third, participants filled in the part of the delivery results they reported themselves. Any deviations from their intended use were documented and analyzed. This combined method allowed us to evaluate the compliance and quality of application usage across the cluster. The REST mobile app includes a built-in troubleshooting and decision support feature designed to assist midwives in early identification and management of maternal health issues. This feature operates on a rule-based algorithm that evaluates input data, such as blood pressure and hemoglobin levels against reference thresholds established by the Indonesian Ministry of Health and WHO guidelines. If an abnormal value is entered, the app automatically flags the entry and provides a visual alert. It then offers a set of recommended clinical actions, such as counseling, closer follow-up, or referral to a higher-level facility. Midwives retain clinical autonomy in making final decisions, but the app serves as a structured guide to enhance consistency and responsiveness in care. This feature was designed to strengthen early detection and ensure standardization in ANC service delivery, particularly in remote or resource-limited settings.

### Study Outcomes

All results were measured at the end of the study after participants from both the control and the treatment group had undergone labor. The success of the intervention was determined from the examination data input in the REST mobile app. Results were compared between the 2 groups. In addition, the effectiveness of the app was assessed using a questionnaire.

#### Primary Outcomes

##### Pregnancy Complications

Pregnancy monitoring using the mobile REST mobile app can prevent complications during a mother’s pregnancy compared to not using the app. Once the trial began, the definition of primary results was refined from “pregnancy complications” to “specific conditions of complications experienced by the mother during pregnancy, such as anemia, hypertension, and chronic energy deficiency.” These changes were made before data analysis and have been documented in the protocol.

##### Methods of Delivery

Using the REST mobile app can identify conditions that further lead to pathological conditions from the beginning of pregnancy monitoring. Pathological conditions that are immediately resolved can facilitate a normal delivery.

##### Birth Weight of the Baby

In addition to monitoring the mother’s condition, using the REST mobile app can help monitor the health of the fetus as well. The group that used the app paid more attention to the development of the fetus and could anticipate that the baby’s birth weight can reach a normal value of ≥2500 g.

##### Antenatal Care Visits

The REST mobile app records how many maternal examination visit are made to health facilities. Mothers with the REST mobile app who did not undergo examinations in accordance with the minimum ANC visit standard of ≥6 received a notification from their midwives to immediately carry out the examination.

#### Secondary Outcomes

Monitoring using the REST mobile app makes pregnant women play an active role in their pregnancy conditions, including recording examination results independently, so that their knowledge of examination results increases. The pregnant women’s perception of the current pregnancy by monitoring using the REST mobile app will be better. Because mothers will easily monitor their condition, they will understand every health development experienced. The app used by midwives and pregnant women for pregnancy monitoring was evaluated by assessing satisfaction with all aspects of use.

In addition to maternal and child health indicators, this study assessed secondary outcomes related to the impact of the REST mobile app on health systems. Specifically, we evaluated midwives’ perceived workload, workflow changes, and time efficiency through qualitative methods, such as structured interviews and focus group discussions. These data will provide insights into how the app integrates into routine care, its acceptability among providers, and any unintended consequences on workload. Findings will inform future scale-up strategies and the refinement of app features to ensure that digital tools support rather than burden health care providers.

### Ethical Considerations

This study received ethical approval from the Medical and Health Research Ethics Committee (MHREC), Faculty of Medicine, Public Health and Nursing, Gadjah Mada University – Dr Sardjito General Hospital (approval number KE-FK-0653-EC-2023). ClinicalTrials.gov reviewed this protocol (NCT05741931). Prior to enrollment, all eligible pregnant women were provided with detailed information regarding the study’s objectives, procedures, potential risks, and benefits. The information was delivered in a clear and understandable manner by trained research staff. Written informed consent was obtained from all participants. For those with limited literacy, the information was read aloud, and a witness was present during the consent process to ensure comprehension and voluntary agreement.

To protect the confidentiality and privacy of participants, all data collected from pregnant women and midwives were handled in accordance with established ethical standards and data protection regulations. Personal identifiers were removed and replaced with unique codes to ensure anonymity.

Digital data collected through the REST mobile app were encrypted during both transmission and storage and securely stored on password-protected servers accessible only to authorized members of the research team. Any paper-based data were kept in locked storage within a secure research facility. Participants were informed about these measures during the informed consent process, and all procedures were approved by the Institutional Ethics Review Board.

Participation in this research was voluntary, without any coercion. All participants received comprehensive information about the research, especially about the intervention that would be administered and the possible benefits and risks. Participants were free to withdraw at any time without any impact on their access to health services or a penalty.

### Data Analysis

Descriptive statistics were used to find out the basic characteristics of the entire study group. For bivariable analysis, the independent *t* test or Mann-Whitney U test, and the chi-square test or the Fisher precise test were applied for categorical results, adjusting for group design, if necessary.

Multivariable analysis was performed using mixed effects models to account for clustering at the level of the ANC class cluster. A mixed effects linear regression model was used for sustained outcomes, such as the number of ANC visits and birth weight. Mixed effects logistic regression models were used for binary outcomes, such as pregnancy complications and delivery methods. All models included random interception to account for variations between clusters and fixed effects for interventions, as well as potential confounding variables, including maternal age, education level, socioeconomic status, obstetric history, and nutritional status. Statistical significance was determined using a 2-headed *P* value of <.05.

For each primary and secondary outcome, we used an estimate of the effect size and its precision (95% CIs). Other analyses, including subgroup analysis and customized analysis, of study results were also carried out. Stata 14.0 was used to analyze the data.

### Data Management

The data collected will be kept confidential for the duration of the study and disposed of after a period of 5 years. The data collection process from the beginning to the end of the study used 2 different methods. In the control group, data were collected using a form that was transcribed into electronic format, and in the intervention group, data were collected using an app and transcribed into electronic format that was stored on a desktop computer. These stored data will be used for further analysis. The individual appointed as the chair of the student project committee at the institute is responsible for overseeing data management.

To address missing or incomplete data, the researchers implemented proactive data validation and monitoring procedures within the REST mobile app. Real-time data entry validation rules were embedded in the app to minimize input errors and prevent incomplete submissions. In cases where data were missing or inconsistencies were detected, follow-up verification was conducted by the research team through direct contact with the respective participants or midwives, as appropriate. For statistical analysis, missing data will be assessed for patterns and mechanisms. Depending on the extent and nature of the missing data, appropriate methods, such as multiple imputation or complete-case analysis, will be used. To ensure overall data quality and accuracy, regular data audits will be performed, and all electronic data will be stored in secure, access-controlled databases with audit trails. These procedures have been designed to enhance data integrity and minimize bias due to incomplete or inaccurate information.

## Results

In July 2023, the REST mobile app was prepared and tested in small community groups, including midwives and pregnant women, through simulation. In September 2023, the app was further developed and refined based on the feedback from these small group trials and then introduced to study participants. A total of 22 ANC class clusters were involved, comprising 22 midwives and 220 pregnant women in Purworejo Regency. These were divided into 2 groups: 11 (50%) intervention clusters using the REST mobile app and 11 (50%) control clusters. The majority of participating pregnant women were aged 20-35 years (n=168, 76.4%), consistent with national demographic trends. Approximately 185 (84.1%) had secondary education (junior and senior high school), and 57 (25.9%) had tertiary education (college graduates). The participants were evenly distributed across economic quintiles, reflecting diverse socioeconomic backgrounds, and most lived approximately 1 km from a health facility.

To provide a clearer overview of both completed and planned study phases, Table 4 presents the planned timeline for each phase of the RCT.

**Table 4:** Planned timeline^a^ for study phases.

Phase	Start	End
Ethical approval	April 2023	April 2024
Staff training/preparation	July 2023	October 2023
Participant recruitment	November 2023	January 2024
Intervention implementation	February 2024	July 2024
Follow-up/data collection	August 2024	February 2025
Data analysis	March 2025	June 2025
Report writing/publication	July 2025	December 2025

^a^Dates are tentative and may be adjusted based on field conditions and recruitment outcomes.

## Discussion

### Summary

The advantages of using mobile apps for pregnancy monitoring vary from app to app as each app provides different features. The results of previous studies related to the use of mobile apps for pregnancy monitoring are considered effective and are grouped into 4 categories:

- Apps that provide information (eg, the Health Gestation app and CHAT). This app provides information that pregnant women need to monitor their pregnancy [4,27].
- Apps used to conduct counseling between midwives and pregnant women through text messages and a voucher component [28].
- Apps that help pregnant women make decisions and provide them with mental support (eg, the Quantitative Innovation in Predicting Preterm Birth [QUiPP] app and SP^+^ [16,29].
- Apps that help pregnant women in managing during their pregnancy (eg, the ImTeCHO health assistant app). This app helps pregnant women perform self-monitoring and understand the condition of pregnancy well [10,30,31].

The REST mobile app is the first app designed to be used by midwives and pregnant women in which the data input by the midwives and pregnant women will be integrated. This app can also be used for comprehensive maternal health monitoring. This study used a cluster RCT design with the hope that the group comparison will be used as a benchmark for the effectiveness of using the REST mobile app.

Evidence from RCTs and systematic reviews indicates that mobile-based reminders and educational messaging can significantly increase ANC class attendance, facility-based deliveries, and maternal knowledge. For example, a cluster RCT in Zanzibar [28] demonstrated that mobile health (mHealth) interventions increased the proportion of women attending 4 or more ANC visits from 31% to 44%. Systematic reviews [32,33] further support these findings, confirming that mHealth tools enhance the uptake of maternal and newborn health services. However, although intermediate outcomes have improved, direct evidence showing a significant reduction in maternal mortality through mHealth interventions remains limited. This is largely due to the rarity of maternal deaths and the need for large sample sizes to detect such effects statistically. Despite this, by strengthening maternal health care behaviors and improving access to services, mobile apps like REST are likely to contribute indirectly to the reduction in maternal and neonatal mortality over time.

The risk identification component of the REST mobile app involves midwives identifying risks from the results of the ANC examination. Evaluation counseling involves app-based counseling from midwives for pregnant women, which makes it easier for pregnant women to make decisions. Systematic monitoring by midwives focusing on 10T includes measuring the weight and height (T1), blood pressure (T2), mid-upper arm circumference (T3), uterine fundal height (T4), tetanus immunization (T5), iron supplement tablets (T6), fetal presentation and fetal heart rate (T7), counseling (including childbirth and postpartum preparation; T8), hemoglobin levels (T9), and providing care as needed (T10) [5]. Troubleshooting involves midwives determining solutions to problems faced by pregnant women in their group.

Although the current version of the REST mobile app is limited to ANC, future development phases aim to expand its functionality to include postpartum and early childhood monitoring. Planned features include digital tools for recording postnatal visits, maternal well-being assessments, breastfeeding tracking, and early detection of postpartum complications. For child monitoring, key components will include vaccination tracking, child growth chart integration, and developmental milestone assessments based on national pediatric guidelines. This expanded scope will support a continuum-of-care approach and further strengthen maternal and child health outcomes through sustained digital engagement beyond pregnancy.

### Strengths and Limitations

We hypothesize that the intervention will result in improvements in the number of ANC visits, a reduction in pregnancy complications, and improved delivery outcomes in the intervention group. However, this study has several limitations typical of eHealth trials. As this study used a cluster RCT design, potential biases, such as selection bias, limited comparability between clusters, and residual confounding despite adjustment, may have influenced the observed effects. Therefore, although the REST mobile app shows promise in supporting maternal health, causality cannot be definitively established, and further research may be needed to confirm these findings in other settings. Next, due to the nature of the intervention, participants could not be blinded, which may have introduced performance and reporting bias. Not all participants used the intervention as intended, revealing challenges related to usability and compliance in digital health trials. Additionally, varying internet access across regions posed a challenge in ensuring consistent results across health app trials. Although a mixed effects model was used to adjust for confounding, unmeasured factors may still contribute to residual confounding. Loss to follow-up could lead to attrition bias if it differs systematically between groups. Lastly, the findings may not be fully generalizable to all pregnant women in Indonesia or other low-resource settings due to the specific characteristics of the study sites and participants.

The REST mobile app installation process must be through a website [26], as the app is not yet available on App Store or Google Play Store. Its use is still limited to the research area, namely Purworejo Regency (Central Java, Indonesia). The output of the research is expected to have an effect on improving maternal and child health, and the app will be further developed more widely to reach national and global levels.

### Conclusion

The cluster RCT study on the use of the REST mobile app aims to compare the welfare of pregnant women. The app used by midwives and pregnant women in the intervention group will help monitor the activities of ANC classes that are routinely carried out by the public health centers. The control group that did not use the app carried out class activities for pregnant women in accordance with implementation standards.

The study highlights the potential of the REST mobile app to enhance maternal health services in rural Indonesia by supporting early risk detection, improving service use, and empowering women through health education. Integration into national health programs is recommended. Findings will be disseminated to local stakeholders through presentations and simplified reports and to national policymakers, professional bodies, and the research community via publications, conferences, and open access platforms.

## Appendix

Multimedia Appendix 1 CONSORT-eHEALTH checklist (V 1.6.1).

Multimedia Appendix 2 Fillable SPIRIT Outcomes 2022 checklist, along with the SPIRIT 2013 checklist.

## Abbreviations

ANC: antenatal care
CONSORT: Consolidated Standards of Reporting Trials
DE: design effect
ImTeCHO: Innovative Mobile Phone Technology for Community Health Operations
mHealth: mobile health
MMR: maternal mortality rate
RCT: randomized controlled trial
REST: Risk Identification, Evaluation Counseling, Systematic Monitoring, Troubleshooting
SPIRIT: Standard Protocol Items: Recommendations for Interventional Trials
TIDieR: Template for Intervention Description and Replication
WHO: World Health Organization

## Data Availability

The datasets generated during this study are available from the corresponding author upon reasonable request.
